# Association of polygenic risk scores, traumatic life events and coping strategies with war-related PTSD diagnosis and symptom severity in the South Eastern Europe (SEE)-PTSD cohort

**DOI:** 10.1007/s00702-021-02446-5

**Published:** 2021-11-27

**Authors:** Heike Weber, Adam X. Maihofer, Nenad Jaksic, Elma Feric Bojic, Sabina Kucukalic, Emina Sabic Dzananovic, Aferdita Goci Uka, Blerina Hoxha, Valdete Haxhibeqiri, Shpend Haxhibeqiri, Nermina Kravic, Mirnesa Muminovic Umihanic, Ana Cima Franc, Romana Babic, Marko Pavlovic, Alma Bravo Mehmedbasic, Branka Aukst-Margetic, Abdulah Kucukalic, Damir Marjanovic, Dragan Babic, Nada Bozina, Miro Jakovljevic, Osman Sinanovic, Esmina Avdibegović, Ferid Agani, Bodo Warrings, Katharina Domschke, Caroline M. Nievergelt, Jürgen Deckert, Alma Dzubur-Kulenovic, Angelika Erhardt

**Affiliations:** 1 Department of Psychiatry, Psychosomatics and Psychotherapy, Centre of Mental Health, Julius-Maximilians-University, Margarete-Höppel-Platz 1, 97080 Würzburg, Germany; 2 Department of Psychiatry, University of California San Diego, La Jolla, CA USA; 3 Department of Psychiatry and Psychological Medicine, University Hospital Center Zagreb, Zagreb, Croatia; 4 Department for Genetic and Biotechnology, International Burch University, Sarajevo, Bosnia and Herzegovina; 5 Department of Psychiatry, University Clinical Center, Sarajevo, Bosnia and Herzegovina; 6 Department of Psychiatry, University Clinical Center of Kosovo, Prishtina, Kosovo; 7 Department of Medical Biochemistry, University Clinical Center of Kosovo, Prishtina, Kosovo; 8 Institute of Kosovo Forensic Psychiatry, University Clinical Center of Kosovo, Prishtina, Kosovo; 9 Department of Psychiatry, University Clinical Center of Tuzla, Tuzla, Bosnia and Herzegovina; 10 Community Health Center, Zivinice, Bosnia and Herzegovina; 11 Department of Psychiatry, University Clinical Center of Mostar, Mostar, Bosnia and Herzegovina; 12 Department of Psychiatry, University Hospital Sestre Milosrdnice, Zagreb, Croatia; 13 Center for Applied Bioanthropology, Institute for Anthropological Researches, Zagreb, Croatia; 14 Department of Laboratory Diagnostics, University Hospital Center Zagreb, Zagreb, Croatia; 15 Department of Neurology, University Clinical Center of Tuzla, Tuzla, Bosnia and Herzegovina; 16 Faculty of Medicine, University Hasan Prishtina, Prishtina, Kosovo; 17 Department of Psychiatry and Psychotherapy, Medical Center-University of Freiburg, Faculty of Medicine, University of Freiburg, Freiburg, Germany; 18 Department of Psychiatry, Max Planck Institute of Psychiatry, Munich, Germany

**Keywords:** PTSD, CAPS, Polygenic risk score, Life events, Coping style

## Abstract

**Objectives:**

Posttraumatic stress disorder (PTSD) is triggered by extremely stressful environmental events and characterized by high emotional distress, re-experiencing of trauma, avoidance and hypervigilance. The present study uses polygenic risk scores (PRS) derived from the UK Biobank (UKBB) mega-cohort analysis as part of the PGC PTSD GWAS effort to determine the heritable basis of PTSD in the South Eastern Europe (SEE)-PTSD cohort. We further analyzed the relation between PRS and additional disease-related variables, such as number and intensity of life events, coping, sex and age at war on PTSD and CAPS as outcome variables.

**Methods:**

Association of PRS, number and intensity of life events, coping, sex and age on PTSD were calculated using logistic regression in a total of 321 subjects with current and remitted PTSD and 337 controls previously subjected to traumatic events but not having PTSD. In addition, PRS and other disease-related variables were tested for association with PTSD symptom severity, measured by the Clinician Administrated PTSD Scale (CAPS) by liner regression. To assess the relationship between the main outcomes PTSD diagnosis and symptom severity, each of the examined variables was adjusted for all other PTSD related variables.

**Results:**

The categorical analysis showed significant polygenic risk in patients with remitted PTSD and the total sample, whereas no effects were found on symptom severity. Intensity of life events as well as the individual coping style were significantly associated with PTSD diagnosis in both current and remitted cases. The dimensional analyses showed as association of war-related frequency of trauma with symptom severity, whereas the intensity of trauma yielded significant results independently of trauma timing in current PTSD.

**Conclusions:**

The present PRS application in the SEE-PTSD cohort confirms modest but significant polygenic risk for PTSD diagnosis. Environmental factors, mainly the intensity of traumatic life events and negative coping strategies, yielded associations with PTSD both categorically and dimensionally with more significant p-values. This suggests that, at least in the present cohort of war-related trauma, the association of environmental factors and current individual coping strategies with PTSD psychopathology was stronger than the polygenic risk.

## Introduction

Posttraumatic stress disorder (PTSD) is obligatorily associated with the exposure to one or more extreme traumatic actual or threatened events, such as death, serious injury or sexual violation and therefore integrated in the chapter of “Trauma- and Stress-Related Disorders” in the current Diagnostic and Statistical Manual DSM-5 (American Psychiatric Association 2013). The exposure includes direct experiencing of the event, but also witnessing as well as repeated confrontation with distressing details of an event. The core characteristics of PTSD are re-experiencing the traumatic event by intrusions and flash-backs, avoidance on cognitive, social and behavioral levels, negative changes in thoughts, hypervigilance, anxiety and other affective disturbances. The symptom duration should exceed 1 month. The lifetime prevalence of PTSD is estimated to 3–7% (Kessler et al. 2012; Wittchen et al. 2011). However, as the disorder is event-dependent, there are clear environmentally driven differences in the prevalence. As such, PTSD prevalence reaches up to 30% in war-affected communities or refugees (Priebe et al. 2010; Blackmore et al. 2020). The course of PTSD varies with chronic persistence of symptoms in 50% of affected subjects leading to tremendous individual burden and high socioeconomic costs (Morina et al. 2014). Moreover, PTSD is often comorbid with other psychiatric disorders, such as depression and substance abuse (Jacobi et al. 2014; Morina et al. 2018).

The etiology of PTSD is complex integrating the interaction of trauma-related and individual factors, such as personality traits and genetics (Nisar et al. 2020). Previous evidence suggests, that genetic variations modulate the consequences of the environmental input and by this contribute to the development of clinical symptomatology (Smoller 2016). As in most other psychiatric phenotypes, genetic basis contributing to higher risk for PTSD is complex, including common and rare risk loci of different effect sizes. Numerous candidate gene association studies in targets related to stress and monoamine systems have been performed in the past in PTSD patients and healthy controls to uncover the genetic background, however, leading to inconsistent results (Maul et al. 2020). In recent years, genome-wide association studies (GWAS) including sample sizes of thousands of patients have been conducted to describe the genetic risk structure to develop PTSD (Gelernter et al. 2019; Nievergelt et al. 2019). However, variations derived from GWAS studies, while detectible and replicable at stringent multiple test corrected levels, explain only a very small amount of the genetic or phenotypic variance most likely due to very small true effect size of each gene related to complex disease (Maher 2015). On the other side, there is evidence that the expected rank of realistic effect sizes is outside of the top associations in typical GWAS combined with the expectation that SNPs with lower p-values of the GWAS test statistics are enriched with true disease-related signals (Zaykin and Zhivotovsky 2005). One methodology to capture the associations of different p-values to one variable is to compute polygenic risk scores (PRS) (Choi et al. 2020). PRS are usually derived from a highly powered discovery sample and then applied to the second stage sample using logistic regression. This method attempts to index all possible influential genome-wide variation and allows to integrate the statistically non-significant true signals. Moreover, PRS provides an estimate of genetic liability to a trait at the individual level, which is important for prediction-related approaches. PRS have been so far successfully applied in a variety of psychiatric diseases, such as schizophrenia, bipolar disorder, anxiety and affective phenotypes, to assess the specific genetic background of a disorder but also to explore genetic overlap between psychiatric diseases (e.g. Forstner et al. 2019; Coombes et al. 2020; Xavier et al. 2018). Recently, genome-wide meta-analyses from the Psychiatric Genomic Consortium (PGC) including a large number of global studies provided PRS for PTSD (Duncan et al. 2018; Nievergelt et al. 2019). Both studies showed a specific genetic component on the development of PTSD, however, shared genetic risk between PTSD and other psychiatric disorders was also repeatedly identified. Thus, while polygenic risk is related to the development of psychopathology after traumatization, studies detecting the magnitude of this risk in cohorts with different traumatization context are at an early stage.

Beyond genetics, additional environmental and individual factors are known to be related to PTSD diagnosis and to moderate PTSD symptoms (Sayed et al. 2015). Previous trauma exposure, cumulative exposure to potentially traumatic experiences and trauma severity show robust evidence of an association with PTSD as independent risk factors and in interaction with genetics (Steel et al. 2009; Kolassa et al. 2010; Karam et al. 2014; Tortella-Feliu et al. 2019). An additional psychological factor associated with PTSD is the ability of the individual to cope with the distress related to trauma. For instance, avoidant coping strategies were positively associated with PTSD symptoms whereas positive coping styles were negatively associated with PTSD in both youth and adult patient cohorts (Vernon et al. 2009; Kyron et al. 2021; Powell et al. 2021). Furthermore, sex is important for several aspects of PTSD (Christiansen and Berke 2020). The prevalence of PTSD in women in cross-national community epidemiological surveys worldwide is nearly twice as high as in men after similar traumatic events (Kessler et al. 2017). Genetic factors might correlate with the sex-related difference in prevalence, as shown in recent candidate and genome-wide association studies in PTSD (Smoller 2016; Duncan et al. 2018). Lastly, age at traumatization might be related to the development of PTSD. Younger age of trauma was shown to be associated with higher risk for a variety of psychiatric conditions lifetime, including PTSD and depression (McCutcheon et al. 2010; Meneses et al. 2021; Veeser et al. 2021). However, how age at trauma is related to the development of PTSD in samples of adult patients is unclear, probably because of other significant moderating factors such as types and severity of trauma as well as additional sociodemographic parameters.

In the present study, we applied PRS to evaluate the heritable genetic liability for PTSD in the South Eastern Europe (SEE-PTSD) study cohort, containing traumatized individuals in the context of the wars between 1991 and 1999. PRS were derived from the European UK Biobank mega-cohort (UKBB) (Sudlow et al. 2015) using weights identical to those in the original studies since the accuracy of PRS estimates are more precise when derived from subjects of similar ancestral background (Martin et al. 2019). The primary hypothesis of the analysis was a significant association of PRS to PTSD as diagnosis and severity of PTSD symptoms. As trauma is a prerequisite for the development of PTSD and coping one of the individual factors possibly moderating the development and course of the disease, we further hypothesized that number and intensity of life events as well as positive and negative coping are correlated with PTSD outcome categorically and dimensionally. In addition, sex and age at war were added to the regression model as possible moderating factors for disease pathology and genetic risk.

## Materials and methods

### Study participants

In total, 747 (68.1% males) inhabitants of South Eastern Europe (SEE) were recruited between 2013 and 2015 at five research centers in Bosnia-Herzegovina (Mostar, Sarajevo, Tuzla), Croatia (Zagreb) and the Republic of Kosovo (Prishtina) under the Stability Pact for the SEE collaborative research study (study design description see Dzubur-Kulenovic et al. 2016), in the context of DAAD (Deutscher Akademischer Austausch Dienst) funded program on the scientific (re)development of successor states of the former Yugoslavia with a priori defined contribution of 5 universities in 3 of 7 successor states. The cohort was recruited to evaluate the (epi)genetic contribution of candidate gene variations to TPSD (Ziegler et al. 2018) and was additionally genotyped using a GWS approach to contribute to the combined analysis within the PTSD Psychiatric Genomics Consortium (Duncan et al. 2018). In the timeframe from 1991 to 1999, most of participants experienced severe trauma related to war, leading in many cases to the development of PTSD symptoms. Diagnosis of current, remitted or no lifetime PTSD, was assessed according to the DSM-IV criteria (Kulenović et al. 2008; Priebe et al. 2010). Participants of all three groups (current, remitted or no lifetime PTSD) underwent the same recruitment process. For participants with no diagnosable DSM-IV lifetime PTSD, no Clinician Administrated PTSD Scale (CAPS) (Blake et al. 1995) were recorded during recruitment. In brief exclusion criteria were for all three groups: age younger than 16 years at time of traumatization, age older than 65 years at time of recruitment, intellectual disability (MMSE < 25), organic or brain trauma related disorders, epilepsy, psychotic and addiction disorders (except from smoking), oncological illness, methylation affected medication (e.g. valproic acid) and 1st- or 2nd-degree relation to an already recruited participant. Additional information on the study design, recruitment process, diagnostic evaluation and other sample characteristics are described in more detail by Dzubur-Kulenovic et al. (2016).

After quality control of the genome-wide genotype data for PRS analysis (see below), the study sample included 658 participants of European ancestry (mean age 49.1 ± 8.0; 66.4% males), comprising 189 patients suffering from current PTSD (mean age 49.9 ± 6.8; 67.7% males), 132 remitted probands with lifetime PTSD symptoms (mean 48.9 ± 8.4; 63.6% males) and 337 healthy volunteers who did never develop PTSD (mean age 48.8 ± 8.5; 66.7% males) (Table 1).

**Table 1:** SEE-PTSD sample characteristics are presented for patients with current PTSD diagnoses, patients remitted from PTSD, combined

Characteristics	Current PTSD	Remitted PTSD	Combined PTSD	Healthy probands
*N* (males/females)	189 (128/61)	132 (84/48)	321 (212/109)	337 (225/112)
Age at war (mean ± SD)	32.33 ± 6.64	31.46 ± 7.96	31.98 ± 7.21	31.11 ± 8.26
Age at inclusion (mean ± SD)	49.85 ± 6.75	48.89 ± 8.40	49.45 ± 7.48	48.81 ± 8.53
CAPS score (mean ± SD)	79.30 ± 20.33	66.16 ± 17.64	73.97 ± 20.31	n.a
CAPS score Cluster B (mean ± SD)	24.37 ± 7.40	21.31 ± 6.10	23.13 ± 7.04	n.a
CAPS score Cluster C (mean ± SD)	31.22 ± 8.89	24.75 ± 7.83	28.59 ± 9.03	n.a
CAPS score Cluster D (mean ± SD)	23.71 ± 6.81	20.1 ± 6.45	22.19 ± 6.92	n.a
Number of life events (mean ± SD)	90.31 ± 37.97	73.49 ± 41.85	83.42 ± 40.40	45.37 ± 44.73
Before war (mean ± SD)	4.30 ± 16.02	1.92 ± 9.70	3.32 ± 13.81	1.46 ± 8.07
During war (mean ± SD)	80.96 ± 31.67	68.42 ± 37.01	75.80 ± 34.47	42.53 ± 43.81
After war (mean ± SD)	5.05 ± 14.56	3.38 ± 13.85	4.37 ± 14.27	1.45 ± 6.23
Intensity of life events (mean ± SD)	39.93 ± 16.95	31.33 ± 13.92	36.38 ± 16.31	14.54 ± 12.60
Before war (mean ± SD)	3.48 ± 5.77	1.77 ± 2.76	2.78 ± 4.84	1.25 ± 2.34
During war (mean ± SD)	30.93 ± 14.00	26.37 ± 13.20	29.05 ± 13.84	11.18 ± 10.32
After war (mean ± SD)	5.48 ± 4.88	3.2 ± 4.18	4.54 ± 4.73	2.09 ± 3.37
Positive coping (mean ± SD)	1.46 ± 0.38	1.56 ± 0.34	1.5 ± 0.37	1.48 ± 0.37
Negative coping (mean ± SD)	1.6 ± 0.40	1.56 ± 0.42	1.59 ± 0.41	1.33 ± 0.42

PTSD patients (current and remitted) and healthy participants. No CAPS scores were recorded for the healthy controls during recruitment

CAPS, Clinician-Administered PTSD Scale; mean ± SD, mean and standard deviation; *N*, total and sex-specific individual counts; n.a., data not available; PTSD, posttraumatic stress disorder

Ethical votes at the participating clinical centers were obtained between 2011 and 2013 on the basis of local translations of an information and consent form designed by the Würzburg center. All participants thus were informed and gave written informed consent according to the principles of the declaration of Helsinki (WMA 2013).

### Psychometric instruments

The psychometric measurements of the PTSD diagnosis, severity, life events and coping are described in detail in the original SEE-PTSD-study papers (Priebe et al. 2010; Dzubur-Kulenovic et al. 2016). The presence of current, lifetime or no PTSD symptoms was determined on the basis of the Mini International Neuropsychiatric Interview (M.I.N.I. 5.0.0) (Sheehan et al. 1998). The severity of PTSD symptoms was rated by means of the Clinician Administrated PTSD Scale (CAPS) (Blake et al. 1995). In order to differentiate the severity of various symptom groups, CAPS subscores were formed for symptom cluster B (persistent re-experiencing of trauma), C (persistent avoidance of trauma associated stimuli) and D (persistent symptoms of arousal). The Life Stressor List (LSL) (Sarason et al. 1978) was applied to provide information on type, frequency and severity of traumatic life events before, during and after war. To assess ways of coping the adapted Hoffman–Lazarus Coping Scale (Arcel et al. 1995) with subscales for social support, confrontation, distancing, self-control, positive reappraisal, planned problem solving, escape-avoidance and accepting responsibility, was used.

### DNA extraction, genotyping, quality control and imputation

Genomic DNA was isolated from frozen venous EDTA-blood by using the FlexiGene DNA Kit (Qiagen, Hilden, Germany) according to the instructions of the manufacturer and stored until further processing at − 80 °C.

Genome-wide genotyping was performed on the Illumina PsychArray v1.1. Genotype quality control and imputation were done according to the RICOPILI pipeline (December 2015b version; (https://academic.oup.com/bioinformatics/article/36/3/930/5545088), as described in detail in Nievergelt et al. (2019). In brief, samples and SNPs were quality controlled based on standard procedures, and only subjects of European ancestry were included in genetic analyses to minimize effects of population structure. Principal components (PC) were calculated using EIGENSTRAT (Price et al. 2006). Genotypes were pre-phased with SHAPEIT2 (Delaneau et al. 2013) and imputed using IMPUTE2 (Howie et al. 2009) based on the 1000 Genome Phase 3 reference data (The 1000 Genomes Project Consortium 2015). Imputed genotypes were converted from probabilities to ‘best guess’ hard calls, where genotypes were called based on highest imputation probability. If highest probability was < 0.8, the genotype call was instead set to missing. Only markers with < 5% missing rate were retained.

### Polygenic risk scoring

PRS were calculated in the SEE-PTSD target samples with PRSice v2.2.11.b (Euesden et al. 2015) using risk allele effect sizes from the non-overlapping UKBB PTSD GWAS (*N*_Cases_ = 10,389 and *N*_Controls_ = 115,799 (Bycroft et al. 2017; The UK10K Consortium 2015; McCarthy et al. 2016; Nievergelt et al. 2019). Summary statistics from the UKBB GWAS were filtered to remove SNPs that were strand ambiguous, had minor allele frequency < 1%, or INFO score < 0.6). SNPs were linkage disequilibrium clumped over a window size of 250 kb and *r*^2^ threshold of 0.1. PRS were generated at multiple *P* value thresholds (*P*_T_ < 0.000001, 0.0001, 0.001, 0.01, 0.05, 0.1, 0.15, 0.2, 0.25, 0.3, 0.35, 0.4, 0.45, 0.5 and 1). PRS were tested for association with PTSD in the target SEE-PTSD dataset using logistic regression adjusted for 5 PCs. For sub-analyses, we used predicted standardized PRS from the predefined *P* value threshold *P*_T_ = 0.15, explaining the strongest trend (Nagelkerke *r*^2^ = 0.006; *P*_likelihood ratio test_ = 0.084) for an association with the PTSD phenotype.

### Statistical analyses

All statistical tests were conducted in R v3.1.3 (R Core Team 2014). For comparison of patients (remitted PTSD, current PTSD or combined (remitted + current) PTSD) and controls in regard to their predicted polygenetic risk, the number and intensity of traumata experienced, positive or negative strategies for coping, as well as the age at war and sex, multivariate logistic regression models were carried out by adjusting each of the examined variables for all other variables (e.g. examinations of PRS on the PTSD outcome were adjusted for the number and intensity of traumata experienced, positive and negative coping, age at war and sex). Dimensional analyses of the same variables with the CAPS score as outcome were performed by linear regression models adjusted for all variables as well. The genetic analyses were hypothesis-free (genome-wide association analysis), therefore, no power calculation was done a priori in the present study. For all analyses the significance level was set at *P* ≤ 0.05. Since this analysis is an exploratory approach, we did not adjust obtained *P* values for multiple comparison either. For better comparability all *β*-values were *z*-standardized.

## Results

**Fig. 1:**
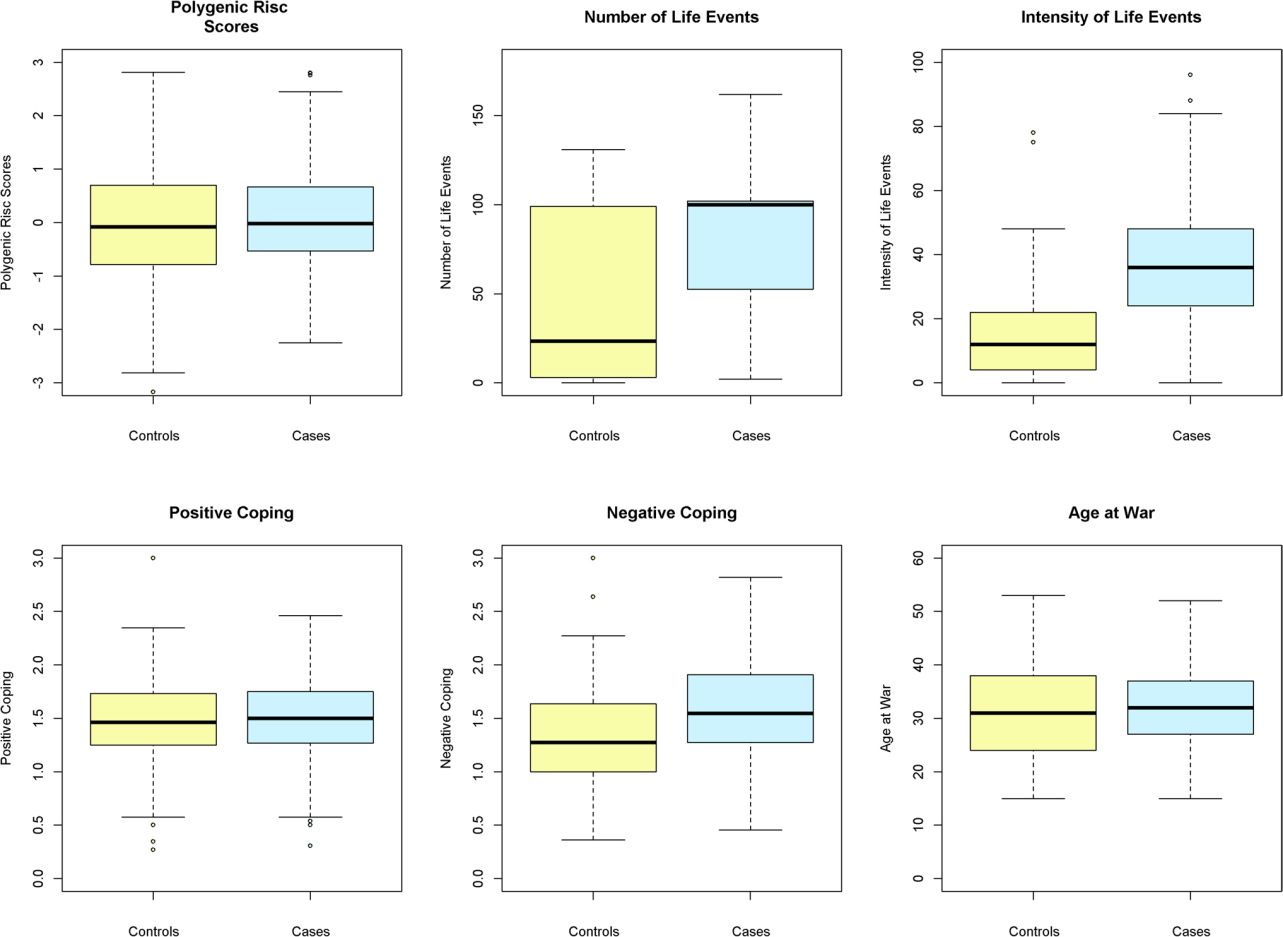
Distribution of polygenic risk scores, the total number and total intensity of life events, positive and negative coping scores and the age at war for the combined patients with diagnosed current and remitted PTSD and controls

To characterize the role of genetic risk and other putative PTSD moderating variables such as traumatic life events, coping style, age at war and sex, we examined their association with the categorical phenotype of PTSD in 189 patients suffering from current PTSD, 132 probands with remitted PTSD and combined (current and remitted PTSD, *N* = 321) in comparison to 337 healthy volunteers (Table 2). In addition to the categorical case–control setting, linear regression analyses were performed on the dimensional CAPS scores in both PTSD patient groups (current and remitted) separately as well as combined (see Table 3). CAPS scores for healthy controls are not available, thus dimensional analyses were restricted to the PTSD patient groups. Further details on sample characteristics are summarized in Table 1. The distribution of values for each examined variable included into the regression model are presented in Fig. 1 for the combined patient group (current and remitted PTSD) and controls.

**Table 2:** Association of the polygenic risk (PRS), number and intensity of traumatic life events, coping strategies, age at war and sex with current, remitted and combined (current and remitted) PTSD diagnosis

Categorical	Current PTSD (*N*_cases_ = 189/*N*_controls_ = 337)		Remitted PTSD (*N*_cases_ = 132/*N*_controls_ = 337)		Combined PTSD (*N*_cases_ = 321/*N*_controls_ = 337)	
	*β*	*P* value	*β*	*P* value	*β*	*P* value
*PTSD diagnosis*						
Polygenic risk scores	0.21	0.108	0.26	**0.040**	0.24	**0.025**
Number of life events	0.23	0.165	0.15	0.327	0.22	0.089
Before war	− 0.06	0.639	− 0.08	0.585	− 0.06	0.595
During war	0.27	0.080	0.18	0.206	0.26	**0.025**
After war	0.08	0.510	0.10	0.427	0.09	0.436
Intensity of life events	1.79	< **2.0 × 10**^**–16**^	1.43	**9.8 × 10**^**–14**^	1.67	<** 2.0 × 10**^**–16**^
Before war	0.63	**7.8 × 10**^**–6**^	0.29	0.087	0.52	**4.6 × 10**^**–5**^
During war	1.66	< **2.0 × 10**^**–16**^	1.42	**6.4 × 10**^**–15**^	1.56	< **2.0 × 10**^**–16**^
After war	0.84	**1.4 × 10**^**–9**^	0.25	0.060	0.64	**5.6 × 10**^**–8**^
Positive coping	− 0.50	**7.5 × 10**^**–4**^	− 0.08	0.612	− 0.28	**0.024**
Negative coping	0.76	**1.7 × 10**^**–5**^	0.52	**0.003**	0.67	**2.7 × 10**^**–6**^
Age at war	0.02	0.867	− 0.06	0.633	0.03	0.808
Sex	− 0.04	0.757	− 0.02	0.905	− 0.02	0.840

*P*-values under the significance threshold of 0.05 were written in bold

PTSD, posttraumatic stress disorder; OR, odds ratio

**Table 3:** Association of the polygenic risk (PRS), number and intensity of traumatic life events, coping strategies age at war and sex with dimensional symptom-related variables recorded by the CAPS questionnaire in all three (current, remitted and combined) PTSD patient groups

Dimensional	Current (*N*_cases=189_)		Remitted (*N*_cases_ = 132)		Combined (*N*_cases_ = 321)	
	*β*	*P* value	*β*	*P* value	*β*	*P* value
*CAPS total*						
Polygenic risk scores	− 0.73	0.603	− 1.04	0.524	− 0.58	0.591
Number of life events	4.42	**0.018**	4.36	**0.012**	5.32	**4.1 × 10**^**–5**^
Before war	− 0.15	0.912	1.93	0.303	0.77	0.484
During war	5.50	**0.007**	4.24	**0.018**	6.02	**1.6 × 10**^**–5**^
After war	0.51	0.649	0.12	0.924	0.25	0.772
Intensity of life events	5.89	**3.4 × 10**^**–4**^	2.09	0.324	5.62	**9.4 × 10**^**–6**^
Before war	3.24	**0.010**	0.61	0.793	3.13	**0.004**
During war	4.68	**0.004**	1.87	0.310	4.41	**3.0 × 10**^**–4**^
After war	3.09	**0.019**	1.17	0.495	3.54	**7.2 × 10**^**–4**^
Positive coping	− 3.54	**0.020**	− 1.78	0.368	− 3.23	**0.007**
Negative coping	4.48	**0.010**	2.74	0.162	4.33	**0.001**
Age at war	− 1.94	0.241	2.85	0.065	0.87	0.450
Sex	2.27	0.117	1.34	0.372	1.62	0.129
*CAPS Cluster B (persistent re-expecting of trauma)*						
Polygenic risk scores	− 0.14	0.780	− 0.05	0.924	− 0.09	0.818
Number of life events	1.03	0.136	1.52	**0.012**	1.45	**0.001**
Before war	− 0.18	0.723	0.92	0.163	0.20	0.602
During war	1.59	**0.032**	1.45	**0.020**	1.75	**2.9 × 10**^**–4**^
After war	0.01	0.990	0.31	0.500	0.08	0.789
Intensity of life events	2.33	**1.3 × 10**^**–4**^	1.40	0.062	2.16	**1.5 × 10**^**–6**^
Before war	1.14	**0.015**	− 0.19	0.813	0.97	**0.012**
During war	2.05	**5.1 × 10**^**–6**^	1.42	**0.028**	1.96	**4.3 × 10**^**–6**^
After war	0.76	0.120	0.00	0.994	0.78	**0.036**
Positive coping	− 0.79	0.156	− 0.46	0.507	− 0.69	0.102
Negative coping	0.53	0.406	0.41	0.551	0.57	0.218
Age at war	− 0.11	0.862	0.71	0.191	0.40	0.318
Sex	0.98	0.070	1.23	**0.021**	1.05	**0.006**
*CAPS Cluster C (persistent avoidance of stimuli associated with the trauma)*						
Polygenic risk scores	− 0.34	0.593	− 0.05	0.948	− 0.06	0.911
Number of life events	2.23	**0.009**	1.25	0.114	2.30	**1.2 × 10**^**–4**^
Before war	0.29	0.623	0.31	0.711	0.49	0.325
During war	2.37	**0.011**	1.33	0.104	2.48	**1.0 × 10**^**–4**^
After war	0.31	0.532	− 0.20	0.730	0.08	0.848
Intensity of life events	1.61	**0.030**	0.76	0.436	1.90	**0.001**
Before war	0.94	0.095	− 0.21	0.844	1.00	**0.044**
During war	1.20	0.099	0.61	0.467	1.40	**0.012**
After war	1.22	**0.037**	0.84	0.273	1.62	**5.9 × 10**^**–4**^
Positive coping	− 1.61	**0.020**	− 0.88	0.332	− 1.61	**0.004**
Negative Coping	1.80	**0.023**	0.98	0.277	1.80	**0.003**
Age at war	− 1.48	0.050	1.45	**0.042**	0.15	0.771
Sex	1.46	**0.029**	0.48	0.491	0.88	0.075
*CAPS Cluster D (persistent symptoms of increased arousal)*						
Polygenic risk scores	− 0.24	0.604	− 0.93	0.117	− 0.36	0.328
Number of life events	1.16	0.060	1.59	**0.012**	1.78	**5.0 × 10**^**–5**^
Before war	− 0.26	0.545	0.69	0.302	0.09	0.801
During war	1.53	**0.024**	1.46	0.025	1.98	**2.4 × 10**^**–5**^
After war	0.19	0.598	0.01	0.976	0.12	0.676
Intensity of life events	1.95	**3.7 × 10**^**–4**^	− 0.07	0.931	1.40	**0.001**
Before war	1.17	**0.005**	1.01	0.225	1.12	**0.002**
During war	1.44	**0.007**	− 0.16	0.810	0.95	**0.021**
After war	1.12	**0.011**	0.32	0.607	1.10	**0.002**
Positive coping	− 1.13	**0.025**	− 0.44	0.545	− 0.93	**0.024**
Negative coping	2.15	**2.2 × 10**^**–4**^	1.35	0.060	1.96	**1.6 × 10**^**–5**^
Age at war	− 0.35	0.527	0.69	0.218	0.36	0.356
Sex	− 0.16	0.738	− 0.37	0.503	− 0.25	0.490

*P*-values under the significance threshold of 0.05 were written in bold

CAPS, Clinician-Administered PTSD Scale

### Polygenic risk

Predictive PRS derived from the UKBB PTSD GWAS were generated in order to test the SEE-PTSD target cohort for genetic PTSD risk loci. The strongest PRS predictions, achieved for the P value threshold *P*_T_ = 0.15 (Nagelkerke *r*^2^ = 0.006; *P*_likelihood ratio test_ = 0.084), were used to examine the association of predicted polygenic risk on the categorical phenotype of PTSD or PTSD dimensions assessed by the CAPS scores (Tables 2, 3).

Categorical regression analyses showed, that predicted polygenic risk did not differ in the current PTSD symptom-specific group from controls, however, was significantly increased in the remitted (*β* = 0.26; *P* = 0.040) and combined (*β* = 0.24; *P* = 0.025) PTSD patients in comparison to the healthy participants (Table 2).

In dimensional analyses including only PTSD patients, predicted polygenic risk was neither found to be correlated with mean total CAPS scores nor with Cluster B, C and D subscores in patients with current, remitted or combined (current and remitted) PTSD symptoms (*β*_all_ < 0.05; *P*_all_ > 0.05), respectively (Table 3).

### Number and intensity of traumatic life events

In addition to the genetic background, we further examined the relationship between traumatic experiences and the development of PTSD. The intensity and number of traumatic experiences were evaluated in total as well as separately before, during and after the war (Tables 2, 3).

The intensity, but not the accumulation of traumatic life events was significantly associated with PTSD diagnosis in all patients (current, remitted and combined) compared to healthy subjects (*β*_all_ > 1.40; *P*_all_ < 1.0 × 10^–13^). The greatest differences between all examined patient samples and controls could be detected for the intensity of traumatization during the war (*β*_all_ ≥ 1.40; *P*_all_ ≤ 1.0 × 10^–14^). Affected patients with current PTSD diagnosis showed in addition a significant association of traumatizing experiences before (*β* = 0.63; *P* = 7.8 × 10^–6^) and after the war than controls (*β* = 0.84; *P* = 1.4 × 10^–9^), which was not the case in the remitted PTSD patient group. That suggests that the resulted association of life events intensity on PTSD diagnosis is probably mainly driven by the patients with current PTSD diagnosis (Table 2).

With increasing number and intensity of traumatic life events, mainly during the war, also a sharp increase in the CAPS total value was observed, especially in patients with current PTSD (number: *β* = 4.42; *P* = 0.018 and Intensity: *β* = 5.89; *P* = 3.4 × 10^–4^) as well as in the combined patient sample (number: *β* = 5.32; *P* = 4.1 × 10^–5^ and Intensity: *β* = 5.62; *P* = 9.4 × 10^–6^), while patients with remitted PTSD symptoms only reached the level of significance with regard to the frequency of trauma experienced (*β* = 4.36; *P* = 0.012). In line with case–control analyses, long-term traumatization seemed to be related to a chronic course of PTSD, which is further supported by the association of higher CAPS scores with higher trauma intensity exclusively in patients with severe current PTSD symptoms before the war (intensity: *β* = 3.24; *P* = 0.010) and after (intensity: *β* = 3.09; *P* = 0.019) (Table 3).

Sub-analysis on CAPS questionnaire showed, that persistent re-expecting of trauma, recorded by Cluster B items, were most strongly associated with increased frequency (*β*_all_ > 1.59; *P*_all_ ≤ 0.032) and intensity (*β*_all_ > 1.42; *P*_all_ ≤ 0.028) of trauma exposure during the war in all three (current, remitted and combined) analyzed patient groups. In contrast, associations on number and intensity of traumatic life events with stronger persistent avoidance of trauma related stimuli assessed by Cluster C items were limited to the current PTSD and combined sample. While in patients with current PTSD symptoms the frequency of traumatic life events during the war was correlated with higher trauma-related avoidance behavior (*β* = 2.37; *P* = 0.011), the intensity of trauma exposure after war was associated with increased avoidance to trauma related stimuli (*β* = 1.22; *P* = 0.037). In addition, persistent symptoms of increased arousal covered by Cluster D items were associated with trauma frequency during the war similarly in patients with current (*β* = 0.04; *P* = 1.53) or remitted (*β* = 1.46; *P* = 0.025) PTSD. However, intensity of trauma exposure was significantly related to persistent symptoms of increased arousal in current PTSD independent of trauma timing before, during or after the war (*β*_all_ > 1.12; *P*_all_ ≤ 0.011; see Table 3).

### Coping strategies

To assess whether and in what way different coping strategies for stress are associated with PTSD, the categorical PTSD, as well as the dimensional CAPS phenotype, were examined in relation to existing positive or negative coping strategies in stressful situations (Tables 2, 3).

Results of the case–control studies showed that specifically negative coping strategies are significantly related to current, remitted or combined PTSD diagnosis in contrast to control subjects (*β*_all_ ≥ 0.52; *P*_all_ ≤ 0.003). Additionally, patients with current PTSD diagnosis used positive coping strategies less often than the controls (*β* = − 0.50; *P* = 7.5 × 10^–4^), which is also reflected in the combined PTSD sample in a low significant group difference (*β* = − 0.28; *P* = 0.024; Table 2).

In agreement with findings from categorical examinations, positive coping strategies correlated with lower and negative coping strategies with higher CAPS scores in the current (*β*_Positive_ = − 3.54; *P*_Positive_ = 0.020; *β*_Negative_ = 4.48; *P*_Negative_ = 0.010) and combined (*β*_Positive_ = − 3.23; *P*_Positive_ = 0.007; *β*_Negative_ = 4.33; *P*_Negative_ = 0.001) patient sample. In particular, sub-analyses showed that positive and negative coping strategies are related primarily to avoidance of trauma-associated stimuli (*β*_Positive_ = − 1.61; P_Positive_ = 0.020; *β*_Negative_ = 1.80; *P*_Negative_ = 0.023) and persistent symptoms of increased arousal (*β*_Positive_ = − 1.13; *P*_Positive_ = 0.025; *β*_Negative_ = 2.15; *P*_Negative_ = 2.2 × 10^–4^; Table 3) predominantly in patients with current PTSD symptoms.

### Age at traumatization and sex

With the exception of a few nominally significant findings in the CAPS sub-analyses, neither age at war nor sex were significantly correlated with the categorical PTSD or dimensional CAPS phenotype (Tables 2, 3).

## Discussion

The present analysis in the SEE-PTSD cohort confirms the assumption of genetic components associated with PTSD by showing significant predicted polygenic risk for PTSD as diagnosis in the remitted and total sample, while no such effects have been found for symptom severity measures. The overall significance of PRS related to PTSD was small.

Previous studies aiming at investigating the relation between polygenic risk and PTSD rendered inconsistent results for different PTSD-related phenotypes. Misganaw et al. used PRS derived from the multiethnic PGC-PTSD Freeze 1 (PGC-PTSD, Duncan et al. 2018) in a cohort comprising veterans of recent wars and showed significant association of PRS with PTSD onset as well as PTSD severity (Misganaw et al. 2019). In a study in European participants involved in World Trade Center (WTC) disaster, PRS from the heterogeneous PGC-PTSD Freeze 2 European analysis including over 60 studies (Nievergelt et al. 2019) did not predict PTSD diagnosis or symptom severity (Waszczuk et al. 2020). However, re-experiencing-PRS derived from the Million Veteran Program (MVP) cohort (Gelernter et al. 2019) significantly predicted total and subscale PTSD symptom trajectory over 18 years clinical monitoring, but not the diagnosis. Interestingly, additional psychiatric PRS from very large PGC meta-analyses, e.g. for generalized anxiety, depression and schizophrenia, were found to be associated with PTSD symptoms severity and long-term course trajectory. In line with our findings, PRS and 9/11 exposure severity were independently associated with PTSD severity. In the WTC study, genetics predicted variance in PTSD more strongly in comparison to exposure severity (Δ*R*2 = 0.022 v. Δ*R*2 = 0.006). One explanation for a lower polygenic risk in our study might be the type of traumatization, which mainly differs in duration as well as frequency possibly likely leading to higher effects of the environmental factors. In line with this, all symptom-related PRS analyses were not significant in our study despite previous evidence of genetic correlation of phenotypic traits with PTSD, such as avoidance, re-experiencing and hyperarousal in the MVP (Stein et al. 2021), in addition to differential types of trauma. In summary, our study supports the polygenic structure of risk for PTSD; however, we suggest for our dataset, that the effects of adverse environment are considerably higher by lowering the effects sizes of PRS. As such, current genetic results are limited by mostly lacking distinction between trauma types in the literature, and additional mechanisms of environmental influence on genetic regulation are discussed, such as epigenetic modifications (Ziegler et al. 2018; Howie et al. 2019; Smith et al. 2020).

In our study, the number and intensity of life events as well as positive and negative coping style showed pronounced associations with PTSD diagnosis and symptom severity. As expected, the number of live events mainly during the war and most highly the intensity of life events independently of timing were associated with overall PTSD severity symptoms in subjects with current and in terms of the frequency also in remitted PTSD. When analysing the symptom subscales, highest effects of war-related life events and a broad correlation of intensity of life events were found for re-expecting of trauma and persistent symptoms of increased arousal. Interestingly, the number of war-related life events was associated with these symptom clusters also in remitted subjects. In addition, while it is generally acknowledged that target trauma severity and frequency are related to increased risk of PTSD, lifetime stressors are also known to be associated with disease development (Bonde et al. 2021). Prior trauma was repeatedly shown to be positively correlated with the onset of PTSD (Lowe et al. 2021). In our study, life event severity exposure after the war was additionally significantly associated with both, current PTSD diagnosis as well as PTSD symptoms, but not the remitted status. These results contribute to the growing body of literature showing that an interaction between ongoing trauma with further life events might significantly explain the trajectory of PTSD over time (Smid et al. 2013; Gargano et al. 2019), leading to the suggestion that individuals once exposed to trauma need additional support to cope with possible further stressors to improve the long-term outcome.

Interestingly, positive as well as negative coping style were related to PTSD diagnostic status and regarding the symptom-level were most highly associated with persistent symptoms of increased arousal and persistent avoidance of stimuli associated with trauma in the current PTSD. Lower level of positive coping behaviour has been identified as predictor for clinically significant increase in PTSD symptoms among previously deployed military veterans (Highfill-McRoy et al. 2021). In contrast, one study in survivors of a major earthquake in China revealed that positive coping, such as adaptive and active coping styles, are not negatively related to PTSD in contrast to maladaptive coping, which shows a positive relationship to PTSD symptoms (Peters et al. 2021). A study in rescue and recovery workers involved in the WTC attack showed maladaptive coping as a consistent factors associated with symptomatic PTSD trajectory (Feder et al. 2016). Additionally, in a recent meta-analysis, Gomez et al. revealed that in adults, negative appraisals about the self-influenced PTSD with high effect sizes (*r* = 0.61) and were more strongly related to PTSD than appraisals about the world or self-blame, independent of single vs. multiple traumas or civilian vs. military population (Gómez et al. 2019). Changes over time regarding the association of PTSD and coping styles were reported, although the results are mixed (La Greca et al. 2013; Karstoft et al. 2015; Powell et al. 2021). Our categorical analyses show that negative coping remained also associated in remitted patients, whereas positive coping was negatively associated in current PTSD only. In regard to symptom severity, coping styles were associated only in the group with current PTSD but not in the remitted individuals which points to less stability of association of coping styles and PTSD after remission. In summary, in addition to increasing evidence in the literature, our results support the involvement of both coping styles on PTSD as disease category but also on persistent arousal and persistent avoidance in subjects with current PTSD. Negative coping was associated with current and remitted PTSD status pointing to a possible long-term change towards maladaptive cognitive strategies with possible future risk for mental disorders. Following this, our results show that specific intervention addressing the cognitive part should be provided to affected, but also to remitted, individuals to improve trauma-related psychopathology on long-term trajectory.

Despite previous evidence of putative sex-related differences on genetics and trauma-related phenotypes, the categorical analysis with PTSD as outcome did not show any significant associations of sex and the dimensional analysis only weak nominal association of sex in remitted patients. This supports the notion that the observed genetic risk in our analysis emerges from common, sex-independent risk loci for PTSD.

Age at war was not associated with categorical PTSD and only weakly associated in the subscale of persistent avoidance of trauma-related stimuli in the dimensional analyses. The literature point to a putative risk of younger age for PTSD (Powers et al. 2014; Lai et al. 2021). Notably, our sample consisted of adult participants with age at war > 16 years which might reduce the contribution of age as predictive factor for PTSD in the present analyses.

Overall, the results from our analysis point to the pronounced association of trauma-related factors on psychopathology in individuals traumatized by war-associated experiences (highest associations with intensity of life events). The epidemiological findings of markedly higher PTSD frequency in such cohorts suggest an environmental “dose”- and “pattern”-effect on psychopathology which might override the contribution of individual genetic risk (Priebe et al. 2010; Briere et al. 2016). The interaction between trauma and genes might follow a U-curve leading to following scenarios: (1) in case of low trauma the PTSD phenotype is driven by genetics, (2) moderate trauma produces a phenotype which is dependent on the genetic background and (3) severe trauma leads to a phenotype less dependent on genetics. An example for such non-linear effects on anxiety-phenotype is the Caffeinen-ADORA2A Genotype-Interaction (Childs et al. 2008). In line with this, a recent structural imaging analysis in combat veterans suggest that changes in hippocampal volume can be primarily attributed to environmental factors, such as stress of combat (Bremner et al. 2020). Taken together, further studies are needed to disentangle the magnitude of environmental and genetic involvement in traumatizing environments.

The biggest limitation of the current study is the modest sample size, which does not allow to test for small effects. However, PRS derived from mega-cohorts have been demonstrated to allow the evaluation of a genetic signal in numerically even smaller studies (Euesden et al. 2015). Generally, in PTSD, as in other complex psychiatric diseases, the genetic and phenotype heterogeneity reduced the ability of GWAS to detect disease risk loci (Duncan et al. 2018). While PRS is aimed to better capture the genetic heterogeneity problem, the phenotypic variance remains, e.g. due to diversity in pre- and existing individual risk factors, trauma exposures, clinical presentation, diagnostic classification and the longitudinal course, which makes deep phenotyping essential (Sanchez-Roige and Palmer 2020). In the present study, we tried to overcome the problem of the temporal and categorical phenotype by subgrouping for the remission status and by including a dimensional symptom level analysis within the PTSD group. However, the latter with a limited range of symptom variance in the patient group yielded no association with PRS score. As discussed above, a possible explanation for the small genetic effect may be that the base PRS UKBB sample is population-based, containing subjects with different types and severity of mainly moderate civil trauma in contrast to the severe traumatic war experiences of the SEE-PTSD-cohort. As such, PRS derived from studies in war-related or military traumatization population might be more informative for the SEE-PTSD-sample. Lastly, the cross-sectional and retrospective nature of our study does not allow any definite conclusions on causal relationships and with regard to temporal relationship only conclusions on PRS versus PTSD, to some degree also to trauma versus PTSD but not to coping style versus PTSD, negative coping styles possibly being a consequence of severe trauma. To resolve this, population-based longitudinal assessment including demographic, psychological, physiological and genetic measures are needed.

In summary, the present results suggest a small but significant polygenic risk predicting PTSD in the overall sample most likely driven by common sex-independent risk loci and thus confirms previous findings. In contrast, the analysis shows highly significant association of traumatizing events and negative coping with PTSD as diagnosis as well as with symptom severity and especially with increased arousal measures in our severely traumatized cohort. This supports the necessity and relevance of therapeutic intervention in severe PTSD versus a restrained approach based on a deterministic concept of etiology.

## Data Availability

Not applicable.
